# Psychological Responses and Strategies Towards the COVID-19 Pandemic Among Higher Education Students in Portugal and Switzerland: A Mixed-Methods Study

**DOI:** 10.3389/fpsyt.2022.903946

**Published:** 2022-05-11

**Authors:** Françoise Schwander-Maire, Ana Querido, Tanya Cara-Nova, Maria Anjos Dixe, Djamel Aissaoui, Zaida Charepe, Derek Christie, Carlos Laranjeira

**Affiliations:** ^1^HES-SO University of Applied Sciences and Arts Western Switzerland, School of Health Sciences Fribourg, Fribourg, Switzerland; ^2^School of Health Sciences of Polytechnic of Leiria, Leiria, Portugal; ^3^Centre for Innovative Care and Health Technology (ciTechCare), Polytechnic of Leiria, Leiria, Portugal; ^4^Center for Health Technology and Services Research (CINTESIS), University of Porto, Porto, Portugal; ^5^Comprehensive Health Research Centre (CHRC), Évora, Portugal; ^6^Institute of Health Sciences (ICS), Universidade Católica Portuguesa, Lisboa, Portugal; ^7^Center for Interdisciplinary Research in Health (CIIS), Universidade Católica Portuguesa, Lisboa, Portugal; ^8^Research in Education and Community Intervention (RECI I&D), Piaget Institute, Viseu, Portugal

**Keywords:** Portugal, Switzerland, COVID-19, psychological impact, higher education, coping strategies, mixed-methods study, students

## Abstract

**Background:**

The COVID-19 pandemic has caused overwhelming changes in individual and community daily-life, resulting from the public health measures implemented to contain it, and also from its psychological and socio-economic consequences. These shifts and consequences impacted the entire population, but some groups are more likely to be affected by these changes, including higher education students.

**Objectives:**

a) to investigate mental health status and its determinants among higher-education students in Portugal and Switzerland; and b) to explore adjustment patterns used by these students to overcome the impact of the COVID-19 pandemic.

**Methods:**

A cross-sectional study with a mixed-methods sequential explanatory design was conducted in two phases. First, an online survey was conducted among higher education students in Portugal and Switzerland, in Portuguese and French respectively. A convenience sampling method was used. Second, some participants from the first phase were invited to participate in four online focus group discussions (two in each country) using a maximum variation sampling method.

**Results:**

The survey was answered by 1,880 students. Portuguese students revealed higher levels of stress and anxiety, but lower depression symptoms and less resilient coping compared to Swiss respondents. Hope was identified as an explanatory variable for mental health symptoms in students from both countries. In the focus groups (*n* = 27), 13 adjustment strategies were found, which were subdivided into three spheres: personal, social, and contextual.

**Conclusions:**

The results suggest that the COVID-19 pandemic had a mild to moderate impact on most of the evaluated mental health variables. Nevertheless, the students reacted and mobilized positive short-term strategies, which need to be reinforced in order to prevent long-term psychological harm. In addition, our results can inform psychosocial interventions to minimize psychological impact, anxiety, depression, and stress due to sanitary crises or other population-wide problems or disasters.

## Introduction

COVID-19 has brought physical, psychological, and social hardship to populations around the world. Arguably, the pandemic represents a “perfect storm” for mental health due to its prolonged and unpredictable character, based on an unfamiliar and invisible danger, and its real threat to lives and livelihoods (1).

Among the “at-risk” population, higher-education students have the dual characteristic of being vulnerable to stress and anxiety, and of potentially being able to embrace new technologies or new situations more readily than other groups. The former is evident from studies that pre-date COVID-19. Before the pandemic, “many students across the globe experienced high levels of anxiety, depressive moods, lack of self-esteem, psychosomatic problems, substance abuse, and suicidality” (2). A recent meta-analysis revealed that the prevalence of depression (39%) or anxiety (36%) among college students greatly increased during the COVID-19 pandemic (3). Another systematic review reported a general prevalence of anxiety in Europe as high as 51%, above that in Asia (33%), but lower than in the USA (56%) (4). In various studies, female gender, social isolation, student status, and low quality of social relations were identified as risk factors for lower mental health (5–7).

Although several studies have focused on the mental health of higher education students, there is a scarcity of studies documenting a holistic portrayal of associations between mental health and protective variables, such as hope and resilient coping. Students seem vulnerable to the negative effects of the pandemic and few studies find significant signs of resilience, such as the use of effective coping strategies (6). Moreover, no study used mixed methods to gain a better understanding of how COVID-19 affects mental health and what coping strategies students may employ in response.

The transactional model of stress by Lazarus and Folkman (8) served as the theoretical underpinning of the current study. It posits mutual interactions between people and their environment and suggests that the stress response is highly influenced by individual appraisal processes (9). This rationale underlies the interplay between physical, psychological, cultural, and social elements and their relationships with mental health, psychological well-being, and social functioning (10). As situations vary between countries, an investigation of the mental health and coping abilities of students under COVID-19 seemed of particular interest, if spanning culturally different settings. This study focuses on students in two middle-sized European countries, Portugal and Switzerland, which in December 2021 had a similar number of registered cases of COVID-19: around 1.3 million, for total populations of 10.3 and 8.6 million inhabitants, respectively (11).

The pragmatic aims of this study were: a) to investigate mental health status and its determinants among higher-education students in Portugal and Switzerland; and b) to explore adjustment patterns used by students to overcome the impact of the COVID-19 pandemic.

## Methods

**Figure 1:**
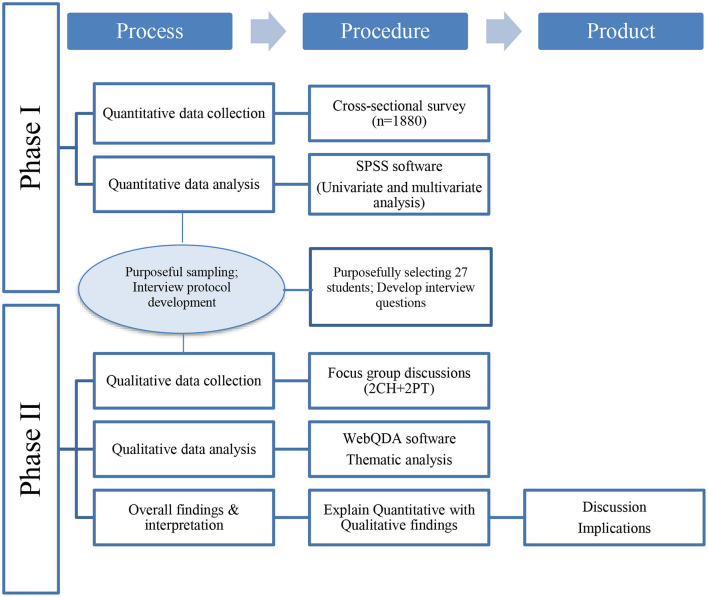
Process flow diagram of the procedures for this sequential explanatory mixed-methods study.

The study followed a mixed-methods approach with a cross-sectional explanatory sequential design, beginning with a quantitative phase that supplied context and participants for the subsequent qualitative phase (see Figure 1). Afterwards, the findings of the quantitative and qualitative phases of the study were combined (12).

The two parts of this international study were as follows:

a) an online survey (quantitative) assessing the mental health status and psychological responses of higher-education students during the COVID-19 pandemic;
b) a Focus Group study (qualitative) discussing the strategies adopted by students to face the COVID-19 pandemic.

Ethical approval to conduct this study was obtained from the Ethics committee in Portugal (CE/IPLEIRIA/22/2020) and Switzerland (swissethics/CER-VD-2020-02889). Informed consent was obtained from all individual participants included in the study.

### Phase I: Quantitative Phase

#### Data Collection

This cross-sectional study involved students from Portuguese and Swiss higher-education institutions. Adults enrolled in education levels above high school, including undergraduate and graduate programs, were considered eligible. The age composition of students in this type of programs is very heterogenous, reflecting actual aging societies and lifelong learning concept. For that reason, no age limit was imposed. Students participating in Erasmus or other mobility programs were excluded. Participants were recruited using convenience sampling. Email contacts of interested participants were recorded in phase I, so they might be invited to participate in the qualitative phase. The total target population of Portuguese and Swiss higher-education students (4 Portuguese institutions and 4 Swiss institutions) was estimated at 61.000 individuals (13). The minimum sample size (*n* = 385) was calculated considering the most conservative scenario (a proportion of 50%), a level of confidence of 95%, and an error margin of 5%. Data were collected between April 2020 and June 2021, via an online survey, sent by the institutions who participated in the study, including:

a) Sociodemographic and health information: gender, age; marital status; level of studies; study area; employment (if applicable); household size; perceived health status (using a scale ranging from 0 [worst] to 100 [best] respondents rated their own physical/mental and overall health); and history of chronic disease.
b) The Depression, Anxiety and Stress Scale-21 (DASS-21) (14), in Portuguese (15) and French (16), to assess psychological distress experienced in the preceding week, using recommended cut-off points (17). Internal consistency (Cronbach's alpha) in this study for depression, anxiety, and stress was 0.92, 0.90, and 0.92, respectively, indicating very good reliability.
c) The Herth Hope Index (HHI), with 12 items designed to measure hope in adults, in Portuguese (18) and French (19). Internal consistency (Cronbach's alpha) in this study was 0.97, indicating excellent reliability.
d) The Brief Resilient Coping Scale (BRCS), a 4-point measure to assess an individual's tendency to cope adaptively (20). The Portuguese version was validated by Pais-Ribeiro and Morais (21), and the French version was translated by Ionescu (22). Cronbach's alpha for this study was 0.79.
e) The Impact of Event Scale-Revised (IES-R) (23), with 22 items describing distress experienced in response to traumatic events. This reliable and valid measure was used in Portuguese (24) and French (25). Internal consistency in the present study was excellent (Cronbach's α = 0.95).

#### Statistical Analysis

Data were analyzed using SPSS Version 28. Descriptive data analysis was performed. Associations and/or differences between variables were determined with univariate and multivariate analyses. A linear regression model was used to evaluate the predictive capabilities of the independent variables with respect to the corresponding decision variables (anxiety, stress, and depression). A 0.05 level of significance was used.

### Phase II: Qualitative Phase

#### Data Collection

Phase II of this study was qualitative research to obtain additional insights into the online survey responses by carrying out Focus Group Discussions (FGDs). Before conducting the FGDs, the authors developed questions based on previous research (13, 26) enabling the acquisition of information regarding the strategies adopted by students to promote better psychological adjustment during the pandemic crisis (13). Participants who completed the anonymous online survey had the option to provide their contact information (e.g., email) if they were interested in participating in the FGDs. Eligible students were selected for the FGDs using a maximum-variation sampling method, creating a heterogeneous purposive sample. Four FGDs (two in each country) were conducted using Microsoft Teams and transcribed in full. In each country, the same non-directive moderating style was used to promote interactions between participants. Each FGD took approximately 60–90 min.

#### Data Analysis

The data were coded and inductively analyzed using a thematic analysis approach (27). WebQDA software was used for qualitative data management (28). The research teams from each country examined qualitative data separately. Swiss and Portuguese data were then merged and analyzed together for common themes and patterns. Themes were identified inductively by two researchers from each country and discussed with the entire research team until consensus was reached. Verbatim quotes from participants were translated from the original into English by a bilingual researcher and used as examples when presenting results. Themes and subthemes were represented using coding trees to illustrate conceptual relationships between the emerging codes. Example extracts from participants were numbered according to FG (1-4), with nationality (PT/CH) in brackets.

#### Validity

The validity of a mixed-method study depends on both the quantitative and qualitative phases. Potential validity threats were minimized by using a large sample size in the quantitative phase, by selecting maximal variety (based on results obtained in phase I) between the participants selected for the FGDs, and by focusing equally on the validity of the two phases (12). To ensure trustworthiness, the researchers were deeply involved in data handling and maintaining rigor during data analysis. The research team adopted a pragmatic stance, integrating the rationale of different methodological approaches (12), and including nurses involved in academic teaching and with practical nursing expertise (AQ, MAD, CL, FSM, TCN, and ZC), as well as a psychologist (DA) and a public health researcher (DC).

## Results

### Quantitative Phase

#### Sociodemographic and Health-Related Characteristics of the Study Sample

Of the 1,880 participants in the survey, 1,522 were from Portugal and 358 from Switzerland (see Table 1). The mean age of participants was 23 years (SD = 6.5) (range 18–59). Most participants were female (88.6%) and single (over 91.2%). Regarding the level of studies, 81.3% (*n* = 1,528) were currently undergraduate students (Bachelor's degree), 38.4% in the field of health area, and 27.6% (*n* = 518) declared being employed. The majority declared no chronic illness, but 13.4% (Switzerland) and 26.3% (Portugal) indicated they lived with elderly or chronically ill people.

**Table 1:** Association between the sociodemographic/health variables and participants country of origin (*n* = 1,880).

		**CH**		**PT**		**X^2^/t**	***p***
		**(*****n*** **= 358)**		**(*****n*** **= 1,522)**			
		***n***	**%**	***n***	**%**		
Age (mean/SD)		23.70	4.64	22.85	6.95	2.197^†^	0.028
Gender	Male	127	35.5	379	24.9	15.939	<0.001
	Female	231	64.5	1,143	75.1		
Marital status	Single	337	94.1	1,388	91.2	5.778	0.123
	Married	17	4.8	118	7.7		
	Divorced	4	1.1	13	0.9		
	widowed	0	0	3	0.2		
Live with elderly or chronically ill people	No	310	86.6	1,121	73.7	26.692	<0.001
	Yes	48	13.4	401	26.3		
Level of studies you attend	Graduation	323	90.2	1,205	79.2	69.210	<0.001
	Post-graduation	17	4.7	15	1.0		
	Master's Degree	16	4.5	154	10.1		
	Other	2	0.6	148	9.7		
Working student	No	196	54.7	1,166	76.6	69.389	<0.001
	Yes	162	45.3	356	23.4		
Study area	Health area	176	49.3	545	35.8	22.258	<0.001
	Other areas	181	50.7	977	64.2		
Diagnosed with a chronic illness?	No	335	93.6	1,285	84.4	19.588	<0.001
	Yes	23	6.4	237	15.6		

† *Student t-test*.

Regarding sociodemographic and health-related variables, statistically significant differences were found between the participants from both countries of origin, except for marital status (*p* = 0.123). A comparison of perceived health status, psychological impact, hope, and coping between the two countries is shown in Table 2. Portuguese respondents reported higher levels of perceived health status compared to Swiss students. However, the severity of psychological impact appeared to be worse in Portuguese students compared to Swiss students (*p* < 0.001). The overall sample had a mean depression score of 7.0 ± 5.6, mean anxiety of 5.2 ± 5.3, and mean stress of 8.2 ± 5.6. Students from Portugal reported higher levels of stress and anxiety, but lower depression levels compared to Swiss respondents (*p* < 0.001; with negligible Cohen's effect size).

**Table 2:** Comparison of perceived health status, psychological impact, hope and coping between the two countries (Portugal vs. Switzerland; *n* = 1,880).

**Variables**	**CH (*****n*** **= 358)**		**PT (*****n*** **= 1,522)**		**t**	***p***	Effect size*
	**Mean**	**SD**	**Mean**	**SD**			
Mental health state, with reference to the last month	42.72	24.76	61.57	21.36	−14.55	<0.001	22.04
Physical health state, with reference to the last month	49.36	22.61	65.09	19.89	−13.09	<0.001	20.44
Overall state of health -with reference to the last month	49.51	21.34	66.86	17.92	−15.86	<0.001	18.62
DASS-Stress	7.54	5.24	8.42	5.75	−2.65	0.008	5.66
DASS-Anxiety	3.61	4.21	5.68	5.48	−6.67	<0.001	5.26
DASS-Depression	9.10	5.52	6.57	5.61	7.67	<0.001	5.59
HHI	32.73	8.19	35.52	5.92	−7.34	<0.001	6.40
BRCS	13.60	2.98	12.50	3.47	5.46	<0.001	3.39
IES-R	21.56	15.90	34.08	19.26	−11.4	<0.001	18.67

* *Cohen's effect size*.

The overall mean hope score (HHI) was 35 (SD = 6.4) (range, 12–48). The BRCS score shows that 1,129 (60.1%) participants had low, 485 (25.8%) moderate, and 263 (14%) high resilient levels of coping. Compared with the Portuguese sample, Swiss participants had significantly higher resilient coping scores, but lower global hope scores (*p* < 0.001 in all cases).

The IES-R global mean score was 31.7 ± 19.3 (range, 0–88). Statistically significant differences between the two groups emerged for this scale, with the Portuguese sample reporting higher psychological impact compared with the Swiss sample (*p* < 0.001; with a small Cohen's effect size).

#### Prevalence and Severity of Mental Health Symptoms and Psychological Impact

The results of the IES-R were as follows (see Table 3): no impact in 37.6%, mild psychological impact in 16.3%, moderate in 5.6%, and severe in 40.5%. Compared to the Swiss sample, Portuguese students had significantly higher scores on the IES-R scale with *p*-values < 0.001.

**Table 3:** Prevalence of mental health symptoms and psychological impact between the two countries (Portugal vs. Switzerland; *n* = 1,880).

		**CH**		**PT**		**X^**2**^**	***p***	**Odds–Ratio (95%)**	***p***
		**(*****n*** = 358*)		**(*****n*** **= 1,522)**					
		***n***	**%**	***n***	**%**				
IES_R	Normal (0–23)	216	60.3	490	32.2	117.620	0.001	3.204	<0.001
	Mild psychological impact (24–32)	63	17.6	243	16.0			(2.526–4.062)	
	Moderate psychological impact (33–36)	12	3.4	94	6.2				
	Severe psychological impact (>37)	67	18.7	695	45.6				
DASS-Stress	Normal stress (0–10)	245	68.8	978	64.3	5.769	0.056	1.206	<0.001
	Mild stress (11–18)	100	28.1	452	29.7			(0.943–1.543)	
	Moderate stress (19–26)	11	3.1	92	6.0				
DASS-Anxiety	Normal (0–6)	279	78.4	962	63.2	36.523	0.001	2.056	<0.001
	Mild anxiety (7–9)	38	10.7	196	12.9			(1.568–2.695)	
	Moderate anxiety (10–14)	30	8.4	227	14.9				
	Severe anxiety (15–19)	9	2.5	107	7				
	Extremely severe anxiety (20–42)	0	0	30	2				
DASS-Depression	Normal (0–9)	194	54.5	1088	71.5	39.391	0.001	0.472	<0.001
	Mild depression (10–12)	59	16.6	173	11.3			(0.373–0.597)	
	Moderate depression (13–20)	96	27.0	246	16.2				
	Severe depression (21–27)	7	2.0	15	1.0				

* *Two missing cases for DASS-21 subscales*.

According to DASS-21, students with clinically significant anxiety were 44% of the total sample. The sum of clinically significant stress was 34.1%. Those with clinically significant depression were 31.7%. Among Swiss participants, respectively 31.2, 21.6, and 45.6% had symptoms of stress, anxiety, and depression above the normal range. More than half of the Portuguese students (64.3%) had normal scores on the stress subscale, but 29.7% were in the mild range, and 6% could be classified as moderate. Regarding anxiety, 63.8% of the total sample had symptoms in the normal range; 12.5% were mild; 15.4% were moderate; 6.6% were severe; and 1.6% were extremely severe. A normal level of depressive symptoms was present in 71.5%, mild in 11.4%, moderate in 16.2%, and severe in 1% of the total sample.

As indicated in Table 3, in almost all the variables under study (except for stress), the COVID-19 pandemic had a more negative impact on Portuguese students than on Swiss students; these differences were statistically significant (*p* < 0.001).

#### Factors Affecting Depression, Anxiety, and Stress Among Students

As shown in Table 4, the perception of mental health state and hope were negatively correlated with stress in both samples. Similarly, perception of mental health state and hope were negatively correlated with depression. In Portuguese students, resilience (β = 0.161, *p* < 0.001) played a protective role relative to depression. Anxiety, perception of mental health (β = −0.98, *p* < 0.001), and hope (β = −0.182, *p* < 0.001) were negatively correlated with depression in the Portuguese sample. In Swiss students, hope and overall state of health were negatively associated with anxiety, while resilience (β = 0.166, *p* = 0.035) was a positive predictor of good mental health. Hope was found to be a significant explanatory variable for all mental health symptoms, in students of both countries.

**Table 4:** Multivariate logistic regression analysis associated with student stress, anxiety, and depression (*n* = 1,880).

		**CH**					**PT**				
**Dependent variable**	**Independent variable**	**β**	***p***	**R^**2**^**	** ${\text{R}}_{\text{Adjusted}}^{2}$ **	**F (p)**	**B**	***p***	**R^**2**^**	** ${\text{R}}_{\text{Adjusted}}^{2}$ **	**F (*p*)**
Stress	State of mental health	−0.098	<0.001	0.361	0.358	98.218	−0.122	<0.001	0.290	0.290	348.80
						<0.001					<0.001
	Hope	−0.134	<0.001				−0.174	<0.001			
Anxiety	State of mental health	N/A					−0.098	<0.001	0.255	0.254	259.82
											<0.001
	Hope	−0.174	<0.001	0.257	0.251	39.941	−0.182	<0.001			
	Overall state of health	−0.059	<0.001			<0.001	N/A				
	Resilience	0.166	<0.035				N/A				
Depression	State of mental health	−0.093	<0.001	0.493	0.490	168.670	−0.092	<0.001	0.443	0.442	402.86
						<0.001					<0.001
	Hope	−0.264	<0.001				−0.452	<0.001			
	Resilience	N/A					0.161	<0.001			

In the Swiss sample, the model showed a predictive capacity ranging from 25.1% for anxiety to 49% for depression symptoms. In the Portuguese sample, explained variance ranged from 25.4% for anxiety to 44.2% for depression. Both models proved satisfactory (29) presenting F values with high statistical significance (*p* < 0.001); and all *t*-test values were significant at p < 0.001.

### Qualitative Phase

The four FGDs relied on a total of 27 participants (Switzerland: 15 participants for 2 FGDs; Portugal: 12 participants for 2 FGDs). Using thematic analysis, 13 subthemes were extracted and classified into three main themes – or spheres – concerning the adjustment strategies adopted by students (see Table 5).

**Table 5:** The coding structure of main themes, subthemes and quoting passages.

**Themes**	**Subthemes**	**Extracted codes**
Personal sphere	Personal growth and adaptability	*FGD1 (PT) “it's a learning process and it's great to see the **ability of human beings to adapt to new circumstances**, but at the same time, it also brings us [...] some insecurities [...].”*
		*FGD2 (CH) “[…] it's mainly the key words of **adaptation, change, fear and awareness** of our environment, but also of ourselves. And then from my point of view, it also allowed us to take **time for ourselves**...”*
	“Window of opportunity” to be involved in academic and professional projects	*FGD1 (PT) “[...] it was also **a window of opportunity**, also because I **managed to get involved in other projects that I would never achieve in terms of geographical distance**.”*
	Focus on Work-life balance	*FGD1 (PT) “**Structuring a timetable is very important […]**. Sometimes we lose a little bit of the notion that **we're working, the brain is working and it's tiring and that we're not taking any time for ourselves**.”*
		*FGD2 (CH) “F**or me I tried to keep a fairly fixed timetable**, I need to know what is going to happen, to have everything planned out [...] **I am doing my routine** like when I was going to school […].”*
	Self-care through the adoption of healthy lifestyles	*FGD1 (PT) “At the beginning of the pandemic, I also **dedicated myself more to sport** and now I ended up becoming a federated athlete, because, really, it was a great escape for me.”*
		*FGD2 (CH) “[.] during the first wave, we had all the time in the world to **do sports** with videos in the garden and all that, […] to cook again, and **I took a lot of time to cook**, which was very good at the time [...].”*
	Being more compassionate to others	*FGD4 (PT) “I felt I shouldn't be sad because **there were people worse than me and I felt I wanted to help**. However, I think I ended up realizing, a bit like my colleague, that our role was to stay at home effectively and try to make others aware of what we could do within our possibilities and within our training [...].”*
		*FGD1 (CH) “For my part, during the whole of the first period, the first wave, **I worked 100% in home care as a back-up because they really needed people**, whether it was to replace people who were COVID-19 positive.”*
	Selecting useful information about the pandemics	*FGD1 (CH) “[...] being nice about the news and social networks, **after a while you don't have to read too much because** (laughs) otherwise I think we'd spend our day getting angry [...] so I think **having stopped watching it has helped me a lot.”***
		*FGD1 (PT) “[...] I felt the issue of **selecting the news** I see was very important. [...] at the beginning, I saw everything and was even afraid to go to the door, and then **I started to select a little bit the news I read to be able to continue to have a normal life**.”*
	Compliance with the sanitary measures	*FGD2 (CH) “I think that during the first wave there were a lot more people who paid attention, who **were more inclined to respect the rules [...]** d**uring the second wave, even now, which still has some [...] that many people are fed up and think that the rules are no longer important [...].”***
		*FGD4 (PT) “**Of course, there are things that stick, hand sanitizing whenever we touch something when we're in public spaces; wearing the mask in public spaces and using it to cover your nose and not put it on your chin [...].”***
Social sphere	New ways of communication – digital networking	*FGD2 (CH) “[…] with my friends, we did quite a lot of “**skypero” as we used to call it, an aperitif in front of Skype**, to take our minds off things, have a laugh.”*
		*FGD1 (PT) “**this group of friends that meets online every day ended up giving me a great escape**. So, we ended up accompanying each other, supporting each other and that was also one of the great pillars for managing to cope with all this stress.”*
	New ways of being physically close	*FGD1 (PT) “[…] although there was no physical contact, in the pandemic, **they were on a balcony and I was down here on time because this contact is very, very, important, at least for me.”***
		*FGD2 (CH) “[...] **I had to recreate a social thing because otherwise I knew I was really going to fall to the bottom**. So, I had to [...] in the end I went for a walk with friends, I invited friends for a drink from time to time, things like that, because otherwise I felt I wouldn't be able to hold on.”*
	Prioritise and valorise “being with my family and friends”	*FGD2 (PT) “**Sometimes we do not give due value to our family [...] what I want is, from now on, to enjoy every moment. Whenever there are opportunities, instead of creating obstacles for us to have these contacts**.”*
		*FGD1 (CH) “But then, now, with my behaviour, I say to myself that I have to enjoy it more. **Maybe I'll make more contact with colleagues, with the family, to make sure they're okay. I think it's a bit of a change from all that [...].”***
Contextual sphere	Adaptation of the “teaching / learning” environment	*FGD2 (CH) “[...] it brought a lot of positive things, also **distance learning**, **for journeys for example, organisation of the days**, sometimes there are those who prefer to work in the evening for example […].”*
		*FGD1 (PT) “I think that the pandemic, in a way, **also made us develop some skills of adaptation to new contexts**. Without doubt! We had to restructure our way of organising ourselves, of living together, of learning, mainly at university level.”*
	School support services	*FGD1 (PT) “The school had a volunteer service. There was a student who also went shopping for me, I also had friends who went shopping for me. ... **It was good to have had that support and also to feel that I wasn't alone.” [...] That kind of support*** (school support service) ***helped me a lot, even to create study methods, methods to decrease anxiety.”***
	Tailored teaching support	*FGD1 (PT) “I was about to give up. Luckily Professor X insisted with me and didn't let that happen. **Luckily, he was always around, he encouraged me, he motivated me, he always met with me, whenever I needed him.”***
		*FGD1 (CH) “[...] **the teachers, they used more direct contacts uh I mean more messages shared on Teams more ... well they made themselves very quickly easily available** on Teams or on the other platforms by mails […].”*

The first theme was related to the personal sphere, where students developed the most adjustment strategies in both countries. They referred to an opportunity for personal growth and adaptability. Portuguese students talked about a “window of opportunity” to be involved in academic and professional projects, although students in Switzerland did not mention this point. Focusing on work-life balance was a strategy mentioned in both countries, mostly by organizing and separating work from private life. Self-care through the adoption of healthy lifestyles was also important and was mostly related to physical activity and/or eating habits. Being compassionate with others emerged as a strategy that helped put things into perspective and remain aware of one's role in helping and protecting others, either directly through volunteer work or indirectly by staying at home and avoiding contacts. Students reported feeling overwhelmed by the quantity of information from different media and felt the need to select the most useful sources. Compliance with sanitary measures was an essential strategy that emerged in both countries with some differences, especially concerning the different waves of the pandemic.

In the social sphere, students evoked new ways of communicating via digital networking to compensate for the lack of physical proximity and stay in safe contact with friends and relatives. Portuguese students mentioned new ways of being physically close, such as communicating through a window or from a balcony, while students in Switzerland found alternative ways of socializing by going for physically distanced walks with friends. A strategy that was greatly discussed in all FGs was the importance of being with family and friends. Students felt more aware of the importance of cultivating these relationships and giving value to time passed with loved ones.

In the contextual sphere, students of both countries talked about the importance of adapting the “teaching/learning” environment. They found themselves in a new situation and had to adapt almost constantly to new ways of studying and learning online. Even in difficult situations, advantages were perceived by some students, such as not having to commute to the university. Tailored teaching support was a significant strategy, especially in maintaining their motivation. Portuguese students brought up that school support services helped them deal with certain difficulties, but this was not mentioned by students in Switzerland.

## Discussion

This study set out to investigate the psychological and mental health responses of higher education students during the COVID-19 pandemic, and the coping strategies patterns they used to manage or deal with the pandemic. We found that 31.7, 44.0, and 34.1% of the participants had depression, anxiety, and stress symptoms, respectively. These rates were lower than in previous studies. For example, a meta-analysis (30) found a higher prevalence of anxiety and depression in pandemic-affected college students. In Switzerland, a survey conducted in April-May 2020 found that 85.8% of surveyed undergraduate students reported symptoms of anxiety, although in most cases the symptoms were mild (63.3%) (31). Another study, with 1,075 Portuguese and Spanish undergraduate students in April-May 2020, revealed high levels of perceived stress (51.9%) (32).

These differences may be related to when data was collected. Higher levels of depression in Switzerland and of anxiety and stress in Portugal suggest timing of data collection is relevant when investigating the prevalence of mental health symptoms. Recent findings seem to indicate that feelings of overwhelming, stress and anxiety were higher in the early months of the pandemic (33, 34). This evidence confirms the relevance of our results from students in Portugal, where data collection started 1 month after the outbreak of the pandemic. However, depressive symptoms, which include feelings of sadness, loss of interest and pleasure in activities, as well as disruptions of daily life, may remain high or even increase during a pandemic due to social isolation and physical distancing (35). This may explain why the Swiss students in our study had higher depressive symptoms: their data collection began 12 months after the start of pandemic.

Our results show that the COVID-19 pandemic had a worse impact on Portuguese than Swiss students. We hypothesize that this may be related to cultural differences. The Portuguese population is predominantly Catholic, with a close-knit family ethic, and experienced the pandemic as markedly disruptive, breaking family ties due to social restrictions, and highlighting rooted fatalistic values (36). Although Switzerland generally scores highly on individualism, Swiss society is also characterized by adherence to rules – a characteristic linked to federalism (a defining attribute of the Swiss political system) and a collective mind-set (37). In such a cultural setting, individuals may attribute high value to the group, thus reducing the personal negative impact of external adverse events. Finally, trust toward scientists is higher in Portugal than in Switzerland (37); such trust may not have been an advantage in the initial phases of a pandemic that caught most scientists by surprise.

Resilience and hope feature prominently in our study, but to our knowledge they have rarely been used when investigating student reactions to the COVID-19 pandemic. Our research stresses the protective role of hope in the mental health status of students. Similar results have highlighted the protective benefits of hope on cognitive abilities in uncertain times (38, 39) and that hope can be a predictor of active coping styles adopted by students (40). Nevertheless, most of our participants had low levels of resilient coping. A possible reason is that the pandemic led to increases in anxiety and depression, but also undermined personal resilience (41).

Our qualitative findings in both countries showed that students developed adjustment strategies mostly in the personal sphere. Confinement and isolation may have made students more introspective, leading to solitude and, in turn, to the development of such strategies. As shown by Höglinger and Heiniger (42), feelings of solitude have been reported as being difficult to manage through the different pandemic waves. The adjustment strategies reported in our study are consistent with coping strategies reported by authors such as Shanahan et al. (43) and Burton-Jeangros et al. (26), who found that frequent use of strategies like keeping a daily routine, reappraisal/reframing, engaging in hobbies and activities and staying connected with others were associated with reduced distress (26, 43). Such approaches are needed to boost resilience factors protecting the individual against psychological distress (6).

Students reported using social media and technology to maintain contact with family members and find information, an important coping strategy to deal with the pandemic lockdown found on other studies (34, 42, 44). This fits well within the concept of digital natives (45): many of today's students feel comfortable with technology from an early age and consider it an integral and necessary part of their lives. Our study supports this concept, especially the positive aspects of social media exposition, such as social connectedness, normalization of behaviors, compliance with government directives and access to support groups. Unsurprisingly, students mainly had a positive attitude toward online teaching and learning activities (46). Nevertheless, some scholars (45, 47) point out negative consequences such as loneliness, psychological distress, sleep deprivation, emotional anxiety, the spread of misinformation, and difficulty to discern facts from fiction.

The findings of this study suggest that the COVID-19 pandemic had a mild to moderate impact concerning most of the evaluated mental health variables. Nevertheless, the students reacted and mobilized positive short-term strategies that should be reinforced in order to protect them from long-term psychological harm. Furthermore, these strategies help students to stay mentally healthy and mitigate delayed-onset post-traumatic stress disorder during the COVID-19 pandemic (48).

### Implications for Practice, Research, and Policy

At a practical level, we suggest that higher education institutions reorganize teaching methods that provide greater consideration for student psychosocial functioning. Goal 3 of the 2030 Agenda for Sustainable Development (49) underlines the importance of new learning methodologies to promote self-care and personal knowledge for good mental health. Other recommendations include increasing social support (e.g., peer support system and professional mediation), which have the potential to alleviate the mental health burden. We also suggest designing or tailoring university-based interventions (e.g. psychoeducation, emotional self-regulation, positive mental health promotion) to address student biopsychosocial needs (50).

At a research level, it is necessary to anticipate future crises and prepare for the transition to the post-COVID-19 world. We need to understand the implications of pandemics on the development of the academic, professional, and personal paths of students. Generally, our understanding of student health must integrate knowledge of their pre-existing health conditions. Future research would benefit from a prospective study with a comparison group that would help understand the longer-term impact of epidemics on the development of psychopathological symptoms. Also, studies investigating longitudinal data on risk and protective factors (e.g., substance use, coping strategies, satisfaction with life, social support, family and peer relationships dynamics) would be helpful.

At a policy level, we believe countries should introduce new forms of mental health support, including information materials (mostly online) and mental health support phone lines. It may be worthwhile to shift some mental health services to a telemedicine format and increase investment in mental health (51). Moreover, the COVID-19 has provided opportunities to build on positive innovations: the flexibility of community-based care, digital healthcare, and the connection of physical and mental health (52).

### Strengths and Limitations

Our results provide a theoretical basis for a broad audience of educators and researchers and may add to a better understanding of the impact of COVID-19 on higher education. Another strength is the mixed-methods research design and the use of valid and reliable instruments. The multicentric online survey provided broad ecological validity, while the focus groups brought explorative depth and consistency, and raised factors that could potentially mitigate some of the negative impacts. Despite these strengths, this study has some limitations. First, data collection did not occur at the same time in each country, because each was submitted to separate ethics committees that did not operate under the same rules or at the same speed. Second, there is an absence of information on pre-existing mental health conditions, which we suggest should be included in future research on the topic. Third, the use of non-probability sampling procedures and self-reporting measures limit the representativeness of the study. Fourth, the sample lacks homogeneous criteria since most of the participants were female with Portuguese nationality. Lastly, our conclusions are based on cross-sectional data, so we are not able to report on changes over time and establish causality.

## Conclusion

Our findings support the idea that the COVID-19 pandemic has a significant impact on the mental health of higher education students. In the future, it would be desirable to focus on positive ways of coping and the potential of hope in this regard. Based on our results, positive coping and hope should be integrated into the standard training of students across all study areas. Indeed, a greater focus on protective factors may be a promising way to prepare students – and others – for future hardships, at individual, community, and societal levels.

## Data Availability Statement

The raw data supporting the conclusions of this article will be made available by the authors, without undue reservation.

## Ethics Statement

The studies involving human participants were reviewed and approved by Ethics Committee in Portugal (CE/IPLEIRIA/22/2020) and Switzerland (swissethics/CER-VD-2020-02889). The patients/participants provided their written informed consent to participate in this study.

## Author Contributions

The study was conceived and designed by AQ, MD, and CL in Portugal and by FS-M and TC-N in Switzerland. Jointly, all five set up an international collaboration, completed the study plan and obtained funding. Data collection and data analysis were carried out by DA, FS-M, and TC-N in Switzerland and by AQ, CL, MD, and ZC in Portugal. DC and CL drafted the first version of the manuscript. All authors contributed to the article and approved the submitted version.

## Funding

This work is funded by CCISP-HES.SO Collaborative Research, through: for Portugal, FCT and Fundação para a Ciência e a Tecnologia, I.P. (UIDB/05704/2020 and UIDP/05704/2020) and under the Scientific Employment Stimulus-Institutional Call—[CEECINST/00051/2018]; for Switzerland, HES-SO Rectorat-Impulsions. Call Swiss-Portuguese Projects (103744/R-PORTUG20-08). Open access funding was provided by the University of Geneva.

## Conflict of Interest

The authors declare that the research was conducted in the absence of any commercial or financial relationships that could be construed as a potential conflict of interest.

## Publisher's Note

All claims expressed in this article are solely those of the authors and do not necessarily represent those of their affiliated organizations, or those of the publisher, the editors and the reviewers. Any product that may be evaluated in this article, or claim that may be made by its manufacturer, is not guaranteed or endorsed by the publisher.

## References

[1] Giannopoulou I Galinaki S Kollintza E Adamaki M Kympouropoulos S Alevyzakis E. COVID-19 and post-traumatic stress disorder: the perfect “storm” for mental health (review). Exp Ther Med. (2021) 22:1162. 10.3892/etm.2021.10596 34504607 PMC8392877

[2] Browning M Larson LR Sharaievska I Rigolon A McAnirlin O Mullenbach L. Psychological impacts from COVID-19 among university students: risk factors across seven states in the United States. PLoS ONE. (2021) 16:e0245327. 10.1371/journal.pone.0245327 33411812 PMC7790395

[3] Li Y Wang A Wu Y Han N Huang H. Impact of the COVID-19 pandemic on the mental health of college students: a systematic review and meta-analysis. Front Psychol. (2021) 12:669119. 10.3389/fpsyg.2021.669119 34335381 PMC8316976

[4] Liyanage S Saqib K Khan AF Thobani TR Tang WC Chiarot CB. Prevalence of anxiety in University students during the COVID-19 pandemic: a systematic review. Int J Environ Res Public Health. (2021) 19:62. 10.3390/ijerph19010062 35010323 PMC8750929

[5] McQuaid RJ Cox SML Ogunlana A Jaworska N. The burden of loneliness: Implications of the social determinants of health during COVID-19. Psychiatry Res. (2021) 296:113648. 10.1016/j.psychres.2020.113648 33348199 PMC9754822

[6] Manchia M Gathier AW Yapici-Eser H Schmidt MV de Quervain D van Amelsvoort T. The impact of the prolonged COVID-19 pandemic on stress resilience and mental health: a critical review across waves. Eur Neuropsychopharmacol. (2022) 55:22–83. 10.1016/j.euroneuro.2021.10.864 34818601 PMC8554139

[7] Xiong JQ Lipsitz O Nasri F Lui LMW Gill H Phan L. Impact of COVID-19 pandemic on mental health in the general population: a systematic review. J Affect Disord. (2020) 277:55–64. 10.1016/j.jad.2020.08.001 32799105 PMC7413844

[8] Biggs A Brough P Drummond S. Lazarus and Folkman's Psychological Stress and Coping Theory. The Handbook of Stress and Health. New Jersey: John Wiley & Sons Ltd. (2017). p. 349–64.

[9] Obbarius N Fischer F Liegl G Obbarius A Rose M. A Modified version of the transactional stress concept according to lazarus and folkman was confirmed in a psychosomatic inpatient sample. Front Psychol. (2021) 12:584333. 10.3389/fpsyg.2021.584333 33746820 PMC7973375

[10] Reupert A. A socio-ecological framework for mental health and well-being. Advances in Mental Health. (2017) 15:105–7. 10.1080/18387357.2017.134290227558710

[11] World Health Organization (WHO). Coronavirus (COVID-19) Dashboard. Available online at: http://www.covid19.who.int (accessed 20 March, 2022).

[12] Creswell J Plano Clark V. Designing and Conducting Mixed Methods Research. 3rd ed. Thousand Oaks: SAGE Publications. (2017).

[13] Querido A Aissaoui D Dixe M Schwander-Maire F Cara-Nova T Charepe Z. Psychological Impacts of the COVID-19 pandemic among portuguese and swiss higher-education students: protocol for a mixed methods study. JMIR Res Protoc. (2021) 10:e28757. 10.2196/28757 34081598 PMC8244726

[14] Lovibond PF Lovibond SH. The structure of negative emotional states - comparison of the depression anxiety stress scales (DASS) with the beck depression and anxiety inventories. Behav Res Ther. (1995) 33:335–43. 10.1016/0005-7967(94)00075-U 7726811

[15] Apóstolo JL Mendes AC Azeredo ZA. Adaptation to portuguese of the depression, anxiety and stress scales (DASS). Rev Lat Am Enfermagem. (2006) 14:863–71. 10.1590/S0104-11692006000600006 17294019

[16] Ramasawmy L. Validation of the “French Depression Anxiety Stress Scales” (DASS-21) and Predictors of Depression in an Adolescent Mauritian Population. (Doctoral Dissertation), Aix-Marseille, France (2015).

[17] Psychology C. DASS 21 Scoring Instructions. Available from: http://comprehensivepsychology.com.au (accessed 20 February 2022).

[18] Viana A Querido A Dixe M Barbosa A. Avaliação da esperança em cuidados paliativos: Tradução e adaptação transcultural do Herth Hope index. Int J Dev Educ Psychol. (2010) 607–16.

[19] Aissaoui D Gronier G Schwander F Cara-Nova T. Validation of the French translation of the Herth Hope Index assessment (HHI-F). Res Squ. (2021). 10.21203/rs.3.rs-753291/v1

[20] Sinclair VG Wallston KA. The development and psychometric evaluation of the brief resilient coping scale. Assessment. (2004) 11:94–101. 10.1177/1073191103258144 14994958

[21] Pais Ribeiro J Morais R. Adaptação portuguesa da escala breve de coping resiliente. Psicol Saúde Doenças. (2010) 11:5–13.

[22] Ionescu S. Traité de Résilience Assistée. Paris: Presses universitaires de France (2011).

[23] Weiss D Marmar C. The impact of event scale – revised. In: Wilson, JP; Keane, TM, editors. Assessing Psychological Trauma and PTSD. New York: Guilford Press (1997).p. 399–411.

[24] Vieira CP Paixão R da Silva JT Vicente HT. Portuguese Version of The Impact of Event Scale – Revised (IES-R). Lisboa: Centro de Investigação em Psicologia Universidade Autónoma de Lisboa. (2020).

[25] Chiasson M Lapierre S Balbinotti MAA Desjardins S Vasiliadis HM. Validation de contenu de la version francophone du questionnaire impact of event scale-revised selon les critères du DSM-5. Prat Psychol. (2018) 24:21–34. 10.1016/j.prps.2017.02.002

[26] Burton-Jeangros C Duvoisin A Lachat S Consoli L Fakhoury J Jackson Y. The impact of the Covid-19 pandemic and the lockdown on the health and living conditions of undocumented migrants and migrants undergoing legal status regularization. Front Public Health. (2020) 8:596887. 10.3389/fpubh.2020.596887 33392134 PMC7772178

[27] Braun V Clarke V. Using thematic analysis in psychology. Qual Res Psychol. (2006) 3:77–101. 10.1191/1478088706qp063oa

[28] Souza FN Costa AP Moreira A Souza DN Freitas F. WebQDA: Manual de Utilização Rápida. Aveiro: UA Editora. (2016).

[29] Pestana MH Nunes Gageiro J. Análise de Dados para Ciências Sociais. 6th ed. Lisboa: Ediçoes Sílabo. (2014).

[30] Chang JJ Ji Y Li YH Pan HF Su PY. Prevalence of anxiety symptom and depressive symptom among college students during COVID-19 pandemic: a meta-analysis. J Affect Disord. (2021) 292:242–54. 10.1016/j.jad.2021.05.109 34134022 PMC8595068

[31] Lischer S Safi N Dickson C. Remote learning and students' mental health during the Covid-19 pandemic: a mixed-method enquiry. Prospects. (2021) 5:1–11. 10.1007/s11125-020-09530-w 33424041 PMC7784617

[32] Laranjeira C Querido A Marques G Silva M Simões D Gonçalves L. COVID-19 pandemic and its psychological impact among healthy Portuguese and Spanish nursing students. Health Psychol Res. (2021) 9:24508. 10.52965/001c.24508 35106391 PMC8801516

[33] Hawes MT Szenczy AK Olino TM Nelson BD Klein DN. Trajectories of depression, anxiety and pandemic experiences; a longitudinal study of youth in New York during the spring-summer of 2020. Psychiatry Res. (2021) 298:113778. 10.1016/j.psychres.2021.113778 33550176 PMC9754702

[34] Sameer AS Khan MA Nissar S Banday MZ. Assessment of mental health and various coping strategies among general population living under imposed COVID-lockdown across world: a cross-sectional study. Ethics Med Public Health. (2020) 15:100571. 10.1016/j.jemep.2020.100571 32838000 PMC7386294

[35] Loades ME Chatburn E Higson-Sweeney N Reynolds S Shafran R Brigden A. Rapid systematic review: the impact of social isolation and loneliness on the mental health of children and adolescents in the context of COVID-19. J Am Acad Child Adolesc Psychiatry. (2020) 59:1218–39.e3. 10.1016/j.jaac.2020.05.009 32504808 PMC7267797

[36] Sousa V. O equívoco da Portugalidade. In: Baptista MM, Franco JE, Cieszynska B. Europa das Nacionalidades. Imaginários, Identidades e Metamorfoses Polí*ticas*. Available online at: http://hdl.handle.net/1822/40123 (accessed February 16, 2022).

[37] Götz FM Ebert T Rentfrow PJ. Regional cultures and the psychological geography of switzerland: person–environment–fit in personality predicts subjective well-being. Front Psychol. (2018) 9:517. 10.3389/fpsyg.2018.00517 29713299 PMC5911505

[38] Yu M Tian F Cui Q Wu H. Prevalence and its associated factors of depressive symptoms among Chinese college students during the COVID-19 pandemic. BMC Psychiatry. (2021) 21:66. 10.1186/s12888-021-03066-9 33514336 PMC7845579

[39] Hicks E McFarland C. Hope as a protective factor for cognitive difficulties during the COVID-19 pandemic. Front Womens Health. (2020) 5:186. 10.15761/FWH.1000186 PMC9197088 35707282

[40] Cheng LN Guo XY Liu HJ Chen Q Cui RS. Hope, death anxiety and simplified coping style scores of nursing students during the outbreak of COVID-19 a cross-sectional study. Medicine (Baltimore). (2021) 100:e27016. 10.1097/MD.0000000000027016 34449474 PMC8389871

[41] Chen T Lucock M. The mental health of university students during the COVID-19 pandemic: an online survey in the UK. PLoS ONE. (2022) 17:e0262562. 10.1371/journal.pone.0262562 35020758 PMC8754313

[42] Höglinger M Heiniger S. The Covid-19 social monitor: a panel study providing evidence about the social and public health impact of the pandemic. Bulletin of the Swiss Sociological Association. (2020) 157:14–9. 10.1371/journal.pone.0242129 33175906 PMC7657546

[43] Shanahan L Steinhoff A Bechtiger L Murray AL Nivette A Hepp U. Emotional distress in young adults during the COVID-19 pandemic: evidence of risk and resilience from a longitudinal cohort study. Psychol Med. (2020) 52:1–10. 10.1017/S003329172000241X 32571438 PMC7338432

[44] Lutz A Gendre A Duperrex O Zürcher K. Projet de Recherche Covidelphi. Promotion de la Santé et Prévention en Période de Pandémie et de Confinement. Lausanne: Unisanté. (2021).

[45] Haddad JM Macenski C Mosier-Mills A Hibara A Kester K Schneider M. The impact of social media on college mental health during the COVID-19 pandemic: a multinational review of the existing literature. Curr Psychiatry Rep. (2021) 23:70. 10.1007/s11920-021-01288-y 34613542 PMC8493361

[46] Wang CY Zhang YY Chen SC. The empirical study of college students' e-learning effectiveness and its antecedents toward the COVID-19 epidemic environment. Front Psychol. (2021) 12:573590. 10.3389/fpsyg.2021.573590 34408688 PMC8366228

[47] Daniels M Sharma M Batra K. Social media, stress, and sleep deprivation: a triple “S” among adolescents. J Health Soc Sci. (2021) 6:159–66. 10.19204/2021/sclm3

[48] Liao Z Zhang X Wang Y Wang T Li X Zhao M. Delayed-onset PTSD and coping strategies of chinese college students during the COVID-19 pandemic. Front Sociol. (2021) 6:734738. 10.3389/fsoc.2021.734738 34778444 PMC8579065

[49] UN, General Assembly. Transforming our world: the 2030. Agenda for Sustainable Development. A/RES/70/1. Available online at: http://www.refworld.org/docid/57b6e3e44.html (accessed 12 February 2022).

[50] Salazar de. Pablo G, De Micheli A, Solmi M, Oliver D, Catalan A, Verdino V, et al. Universal and selective interventions to prevent poor mental health outcomes in young people: systematic review and meta-analysis. Harv Rev Psychiatry. (2021) 29:196–215. 10.1097/HRP.0000000000000294 33979106

[51] OECD. Tackling the Mental Health Impact of the COVID-19 Crisis: An Integrated, Whole-of-Society Response. Available online at: https://www.oecd.org/coronavirus/policy-responses/tackling-the-mental-health-impact-of-the-covid-19-crisis-an-integrated-whole-of-society-response-0ccafa0b/ (accessed 20 March 2022).

[52] McCartan C Adell T Cameron J Davidson G Knifton L McDaid S. A scoping review of international policy responses to mental health recovery during the COVID-19 pandemic. Health Res Policy Syst. (2021) 19:58. 10.1186/s12961-020-00652-3 33823855 PMC8022299

