# Erratum: Psychological responses and strategies towards the COVID-19 pandemic among higher education students in Portugal and Switzerland: A mixed-methods study

**DOI:** 10.3389/fpsyt.2023.1185369

**Published:** 2023-03-24

**Authors:** Frontiers Production Office

**Affiliations:** Frontiers Media SA, Lausanne, Switzerland

**Keywords:** Portugal, Switzerland, COVID-19, psychological impact, higher education, coping strategies, mixed-methods study, students

An omission to the funding section of the original article was made in error. The following sentence has been added: “Open access funding was provided by the University of Geneva.”

The original article has been updated.

